# Licensed Anti-Microbial Drugs Logical for Clinical Trials against Pathogens Currently Suspected in Alzheimer’s Disease

**DOI:** 10.3390/antibiotics10030327

**Published:** 2021-03-20

**Authors:** Leslie C. Norins

**Affiliations:** Alzheimer’s Germ Quest, 4301 Gulfshore Blvd. N., Suite 1404, Naples, FL 34103, USA; leslie.norins@alzgerm.org; Tel.: +1-239-649-1346

**Keywords:** Alzheimer’s disease, dementia, beta-amyloid, germ theory, drug development, clinical trials, herpes, spirochetes, *Chlamydia pneumoniae*, *Porphyromonas gingivalis*, toxoplasma, mycobacteria

## Abstract

There is now considerable evidence that several infectious agents (viruses, bacteria, or parasites) may play a contributing role in the development of Alzheimer’s disease (AD). The six primary suspects are herpes viruses, spirochetal bacteria, *Chlamydia pneumoniae*, *Porphyromonas gingivalis*, mycobacteria, and toxoplasma parasites. Also, some of the antimicrobial and antiviral agents that are used to treat them have shown promise for AD interventions. I describe this evidence and assert it is now time to accelerate clinical trials of these existing drugs, already federally approved, to determine if such treatments can delay, halt, or reverse AD.

## 1. Introduction

It is estimated that 44 million people worldwide have Alzheimer’s disease (AD) or a related form of dementia. In the United States alone, nearly 6 million people have a diagnosis of AD [1]. Furthermore, since the world faces a dramatically increasing population of individuals aged 60 years or older, the number of AD and dementia diagnoses will only continue to grow. Thus, AD represents a major public health concern. Despite having been previously identified as a major research priority, the necessary clinical trials have fallen short, billions of dollars have been spent, and little progress has been made in drug development during the last decade [2]. The brains of patients with Alzheimer’s disease are classically characterized by two hallmark features, beta-amyloid (Aβ)-containing plaques and neurofibrillary tangles, which are composed of hyperphosphorylated forms of the microtubule-associated protein tau [3]. However, there is now growing evidence that several infectious agents are also suspected of contributing to AD, notably through their interactions with Aβ plaques through inflammatory processes.

The ‘germ theory of disease’ dates back to the 1800s [4], but there is more recent evidence that certain infectious agents may be contributing factors to the pathogenesis of AD. For instance, herpes simplex virus 1 (HSV1)-carrying individuals have an increased risk of later developing AD [5]. Further, antiviral medications against the herpes virus have been associated with a decreased risk of developing dementia [6].

There are now several drugs that have been approved for the treatment of infectious diseases by the United States Food and Drug Administration (FDA) which also have been associated with reductions in AD risk or disease progression when used off-label. The infectious agents most often associated with AD are herpes viruses, spirochetes, *Chlamydia pneumoniae*, *Porphyromonas gingivalis*, mycobacteria, and toxoplasma parasites. Here, I present evidence for the relationships between each of these infectious diseases and AD, as well as illustrate the potential use for antiviral and antimicrobial agents for its therapy. I further assert that it is now time to start clinical trials of existing federally approved drugs against each of the top six infectious suspects to determine if such treatments can delay, halt, or reverse the progression of AD.

## 2. Infectious Agents-Six Known Suspects for Alzheimer’s Disease

### 2.1. Herpes Viruses

There are a series of epidemiological studies, as well as genetic research, that have implicated herpes viruses (most notably HSV1) in AD. Albeit primarily correlational, the evidence for this association in human populations is compelling. Individuals who carry HSV1 are at an increased risk of developing AD later in life [7]. This is particularly true for those who carry both HSV1 and the type four allele of the apolipoprotein E gene (APOE-ε4) [8]. This allele has a longstanding association with AD risk in human populations [9]. One report demonstrated that HSV1 infection of cell cultures upregulated microRNA-146a, which is associated with altered immune responses; however, treatment with the antiviral agent acyclovir attenuated the neuroinfectious actions of HSV1 [10]. Altered innate immune responses are considered by many to be associated with both the APOE-ε4 gene and AD progression [11,12]. Importantly, animal research has shown a causal role for the APOE-ε4 genetic variant in the accumulation of Aβ protein [13].

Aβ protein deposits in the brain (also known as plaques) are a hallmark feature of AD [13]. In fact, some researchers have postulated that Aβ is produced in response to HSV1 viral infection and is actually an antimicrobial peptide [14,15]. Research has also shown that Aβ tends to accumulate in brain cells (both using in vitro and in vivo methodology) that have specifically been infected with HSV1 [16,17]. Additionally, in post-mortem AD brain tissue, a significant proportion of HSV1 DNA was associated with Aβ plaques [18]. Moreover, in one study, when the antiviral drugs acyclovir, penciclovir, and foscarnet were used to treat HSV1-infected cell cultures, Aβ protein levels decreased [19]. Taken together, the above data suggest that antivirals which have been effective for the treatment of HSV1 could potentially impact AD progression, primary through interactions with Aβ protein accumulation and the resultant plaques that form.

### 2.2. Spirochetes 

Spirochetes is a group of spiral bacteria that causes infections such as syphilis and Lyme disease. One of the infectious agents from this group most strongly associated with AD is *Treponema pallidum* (*T. pallidum*). *T. pallidum* is the microbe responsible for syphilis, and has been linked to dementia, shrinkage of the brain’s cortex, and deposition of Aβ protein [20]. Periodontal disease, which is characterized by the presence of various species from the genus *Treponema* (*T. pectinovorum*, *T. amylovorum*, *T. lecithinolyticum*, *T. maltophilum*, *T. medium*, and *T. socranskii*) has also been associated with AD in epidemiological work [21]. Other spirochetes have been linked to AD as well. For instance, in 1987, MacDonald and Miranda [22] first noted the presence of Lyme disease-causing *Borrelia burgdorferi* (*B. burgdorferi*) in the brain of an AD patient. Later, it was shown that *B. burgdorferi* causes in vitro glial and neuronal cell cultures to accumulate Aβ in a manner similar to that observed in AD patients [23]. However, it is important to note that some of these links may indeed be solely correlational.

One theory on the role of these bacteria in AD is that spirochetes make biofilms that activate the innate immune system [24]. When toll-like receptor 2 responds to the production of these bacterial biofilms, inflammatory cytokines such as tumor necrosis factor-alpha (TNF-α) and the transcription factor nuclear factor kappa B (NF-κB) are activated. These factors cause not only tissue damage, but also produce Aβ. This same researcher also postulates that treatment with a bactericidal antibiotic (such as penicillin) with a concurrent biofilm disperser (such as rifampin) may help halt AD progression, but only if given early in the course of the disease. In fact, Allen [24] suggests a very specific treatment regimen for early dementia that consists of penicillin (administered intramuscularly) at 1.2 mu biweekly for 3 doses, probenecid at 500 mg bid (to increase the concentration of penicillin), citalopram at 20 mg daily, and rifampin at 500 mg bid. 

Preliminary clinical studies indeed support Allen’s general idea. For instance, in a placebo-controlled clinical study conducted in Canada, oral daily doses of doxycycline (200 mg) and rifampin (300 mg for 3 months) were administered to over a hundred patients with probable AD, and significant therapeutic benefits were noted in cognition and dysfunctional behaviors [25]. Drugs not called “antibiotics”, but which have antibiotic-like action, can also be worthwhile for investigation. For instance, the anti-alcoholism drug disulfiram (which works, at least in part, to break down biofilms) has been recently used for the treatment of Lyme disease. Expanding these clinical trials to include AD is a logical next step [26].

### 2.3. Chlamydia Pneumoniae

Similar evidence has been observed in studies of *Chlamydia pneumoniae* (*C. pneumoniae*)—a bacteria known for commonly causing both upper and lower respiratory infections. This bacteria has been found in post-mortem brain tissue of AD patients [27,28]. Importantly, research has demonstrated a causal role of *C. pneumoniae* in pathological features of AD. Specifically, in one study *C. pneumoniae* was delivered intranasally to wild-type laboratory mice, and Aβ plaques subsequently formed as a result [29]. 

Treatments for *C. pneumoniae* may also be useful in the fight against AD. The antibiotic rifampin has been shown to not only treat the infections caused by *C. pneumoniae*, but it also prevents accumulation of Aβ in cell cultures [30]. Similarly, another antibiotic agent against *C. pneumoniae*, tetracycline, has been shown in vitro to both inhibit Aβ deposit formation and disassemble the pre-formed fibrils that are also associated with AD [31]. It is noteworthy that again we see this relationship between an infectious agent and Aβ which can be ameliorated through the use of drugs that target the microbial suspect.

### 2.4. Porphyromonas Gingivalis 

*Porphyromonas gingivalis* (*P. gingivalis*) is another periodontal disease-causing oral bacterium that is linked to cognitive impairment, dementia, and AD [32,33]. One genome-wide association study (GWAS) has demonstrated that genes of the *P. gingivalis* interactome were significantly enriched in genes that are related to cognitive disorders, AD, and dementia [34]. 

Periodontal disease has also been linked to higher Aβ load in cognitively normal, aged individuals who do not have AD [35]. Further, in one study, oral *P. gingivalis* infections in mice increased production of Aβ within the brain. In this same study, small-molecule inhibitors targeting gingipains were developed and subsequently used. Gingipain inhibition both reduced P. gingivalis infection, Aβ production, and TNF-α expression, and rescued hippocampal cells, suggesting that gingipain inhibitors could potentially be used in the treatment of AD [36]. This group’s small-molecule inhibitor of gingipains (COR388) has already passed Phase 1 clinical trials and is now running a Phase 2/3 study to determine if this drug can improve cognition in people with AD [37]. However, more studies and clinical trials like these are necessary.

### 2.5. Toxoplasma

Toxoplasma (or toxoplasmosis) is a disease that results from infection with the *Toxoplasma gondii* (*T. gondii*) parasite. This parasite can inhabit the human body for a long time, sometimes with no effects, but an immune system challenge can trigger toxoplasma illness and serious health problems [38]. One meta-analysis demonstrates that *T. gondii* is, in fact, another external risk factor for the development of AD [39]. Rodent research has shown that *T. gondii* induced signs of AD in mice, including anxiety-like behaviors, altered social recognition memory, and deficits in spatial memory [40]. Another study found that *T. gondii* infection potentiated memory impairments via a neuroinflammation pathway in a BALB/c mouse model of AD [41]. This particular mouse model of AD-like impairment was created by injecting Aβ into the hippocampus. Thus, again we see evidence that infectious agents may instigate AD through their interactions with both inflammation and Aβ plaques.

In one hallmark study, anti-parasitic drug (nitazoxanide) treatment improved learning and memory impairments in a mouse model of AD that genetically targets the amyloid precursor protein [42]. However, while this association between the *T. gondii* parasitic infection, AD, and amyloid proteins appears to be scientifically supported at a basic science and pre-clinical level, there has been little clinical research into the use of antiparasitic drugs in the treatment of AD. This may be partially due to some generic antiparasitic agents (notably, pyrimethamine) being inaccessible to researchers for many years due to the high costs imposed by pharmaceutical companies [43].

### 2.6. Mycobacteria

Finally, we have mycobacteria. They represent an entire genus of bacteria (nearly 200 species), many of which have been known to cause infectious diseases such as tuberculosis and leprosy in humans. These bacteria are also strongly associated with AD. In fact, pharmaceutical agents against mycobacteria have been shown to decrease AD risk. In one study of aged patients treated with rifampicin for mycobacterial infections, this antibiotic was found to decrease the chances of later developing AD, but only if the drug was taken at a dose of at least 450 mg daily for one year [44]. In another recent study, Bacillus Calmette-Guérin, which is a vaccine against tuberculosis, was found to decrease the incidence of AD development in a sample of people who had it instilled into their bladders as a treatment for bladder cancer. The researchers believed that these results would generalize to larger populations [45]. Notably, the mycobacterium responsible for tuberculosis forms amyloid-like fibrils both in vitro cell cultures as well as in vivo [46].

While there have been conflicting reports on the interaction between the mycobacteria responsible for leprosy and AD, an interesting new paper may shed light on this relationship. Specifically, researchers in Korea administered the antibiotic/anti-inflammatory drug known as dapsone at 100 mg once a day from 2010 to 2015 for the treatment of mild cognitive impairment (MCI). However, because of drug production changes in Korea, the dapsone regimen was discontinued, and the patient who served as the case example subsequently developed AD. Then, in 2018, the physicians were able to restart the dapsone regimen and the patient reverted from AD back to MCI after one year of treatment. The researchers attributed the positive effects of this drug to its anti-inflammatory actions [47].

## 3. Conclusions

In short, there is compelling evidence to suggest that select viruses and microbes are linked to the development and progression of AD. Despite this, there is only limited research to suggest that the treatments against such infectious agents have the potential to halt, delay, or reverse AD. How these drugs affect Aβ plaque deposits is likely a major contributing factor to their therapeutic potential. Interestingly, Aβ itself may in fact be an antimicrobial peptide in the immune system [48]. Inflammation is another important contributor to AD; therefore, anti-inflammatory drugs may also prove to be useful for the treatment of this disorder [49]. 

It is likely that one of the primary route by which these microorganisms impact Aβ and AD are via inflammation processes, notably inflammatory cytokines in the brain. The microbiome has also been shown to play a critical role in the development of dementias recently. In fact, it was recently shown that pathological features of Parkinson’s disease may start via protein accumulation starts in the gastrointestinal system and ascends to the brain via vagal fibers [50]. The microbiome of patients with AD is also significantly altered compared to non-AD control subjects [51]. However, more research is needed on this topic in how it relates to the microorganisms discussed herein and AD.

Despite this growing body of literature to support infectious agents’ participation in AD, few clinical trials are currently underway to test this hypothesis. As mentioned previously, the gingipain inhibitor known as COR388 is currently in Phase 2/3 clinical trials [37]. Another drug currently in Phase 2 clinical testing in the United State is the antiviral valacyclovir. In this 78-week trial, patients are being given valacyclovir at an oral dose of 4 g daily (after titration) in order to slow cognitive decline [52].

There are many FDA-approved drugs for the treatment of the six known infectious suspects of AD. New drug development takes much time and money, but with these already-approved compounds the clinical trial process can be quickly started. Thus, with economies of time and cost, we could potentially find a quicker way to halt, delay, or even reverse AD.
